# Using HSV-1 Genome Phylogenetics to Track Past Human Migrations

**DOI:** 10.1371/journal.pone.0076267

**Published:** 2013-10-16

**Authors:** Aaron W. Kolb, Cécile Ané, Curtis R. Brandt

**Affiliations:** 1 Department of Ophthalmology and Visual Sciences, School of Medicine and Public Health, University of Wisconsin-Madison, Madison, Wisconsin, United States of America; 2 Department of Botany, University of Wisconsin-Madison, Madison, Wisconsin, United States of America; 3 Department of Statistics, University of Wisconsin-Madison, Madison, Wisconsin, United States of America; 4 Department of Medical Microbiology and Immunology, School of Medicine and Public Health, University of Wisconsin-Madison, Madison, Wisconsin, United States of America; 5 McPherson Eye Research Institute, University of Wisconsin-Madison, Madison, Wisconsin, United States of America; Midwestern University, United States of America

## Abstract

We compared 31 complete and nearly complete globally derived HSV-1 genomic sequences using HSV-2 HG52 as an outgroup to investigate their phylogenetic relationships and look for evidence of recombination. The sequences were retrieved from NCBI and were then aligned using Clustal W. The generation of a maximum likelihood tree resulted in a six clade structure that corresponded with the timing and routes of past human migration. The East African derived viruses contained the greatest amount of genetic diversity and formed four of the six clades. The East Asian and European/North American derived viruses formed separate clades. HSV-1 strains E07, E22 and E03 were highly divergent and may each represent an individual clade. Possible recombination was analyzed by partitioning the alignment into 5 kb segments, performing individual phylogenetic analysis on each partition and generating a.phylogenetic network from the results. However most evidence for recombination spread at the base of the tree suggesting that recombination did not significantly disrupt the clade structure. Examination of previous estimates of HSV-1 mutation rates in conjunction with the phylogenetic data presented here, suggests that the substitution rate for HSV-1 is approximately 1.38×10^−7^ subs/site/year. In conclusion, this study expands the previously described HSV-1 three clade phylogenetic structures to a minimum of six and shows that the clade structure also mirrors global human migrations. Given that HSV-1 has co-evolved with its host, sequencing HSV-1 isolated from various populations could serve as a surrogate biomarker to study human population structure and migration patterns.

## Introduction

Herpesviruses are large, enveloped double stranded DNA viruses with genomes that range in size from 124–295 kilobases. The alphaherpesvirus subfamily is characterized by the capacity to establish latent infections in the sensory nerve ganglia. Previous phylogenetic studies have shown that herpesviruses have co-evolved with their hosts. [1] Herpes simplex viruses type 1 (HSV-1) is a member of the alphaherpesviruses and has a genome size of approximately 152 Kb. HSV-1 causes oral mucocutaneus lesions as well as keratitis and encephalitis and is a significant human pathogen. [2], [3] Animal studies in mice have shown that HSV-1 disease severity relies on three factors; innate host resistance, host immune response and viral strains. [4], [5], [6], [7], [8], [9], [10], [11], [12], [13], Neurovirulence studies with different viral strains in infected mice show that disease severity varies from no disease to lethal encephalitis. [18], [19] Further phylogenetic and genomic analysis of viral strains may aid in understanding the genetic aspects virulence.

Previous studies of HSV-1 phylogeny have analyzed viral strains from primarily one geographic region; Europe or North America with modest sample numbers. Phylogenetic analyses with single genes [20], [21], [22] or with small numbers of genomes [23] have consistently yielded a three clade pattern. However, phenotypic analysis using single genes or small clusters of genes may not present an accurate picture of relationships due to recombination. More accurate information on genetic relationships requires the use of whole or nearly complete genomes. Recently, next-generation sequencing techniques have been used to sequence several HSV-1 genomes [23], [24], [25] with more being directly deposited into GenBank. Currently complete, or nearly complete genomic sequences are available from North America, Europe, East Asia and Eastern Africa. The goal of this study was to examine the phylogeny of the strains as well as look for evidence of recombination. The resulting analysis revealed a minimum six clade structure for HSV-1, as well as a topology based on geographic origin of the isolate. Inspection of the phylogenetic data presented here along with previous estimations of HSV-1 substitution rates suggests a rate of approximately 1.38×10^−7^ subs/site/year. Recombination analysis showed evidence of both inter- and intra-clade recombination.

In this study, for the first time a global sampling of HSV-1 strains has been used for phylogenetic analysis and supports the conclusion that HSV-1 strains have co-migrated with their human hosts, leading to geographically separated clades. The recent demonstration that multiplex sequencing of HSV-1 genomes is feasible [23] significantly reduces the cost per genome and using HSV-1 as a surrogate biomarker would reduce the cost and facilitate studies of human migration.

## Materials and Methods

### Distance Analysis

**Figure 1:**
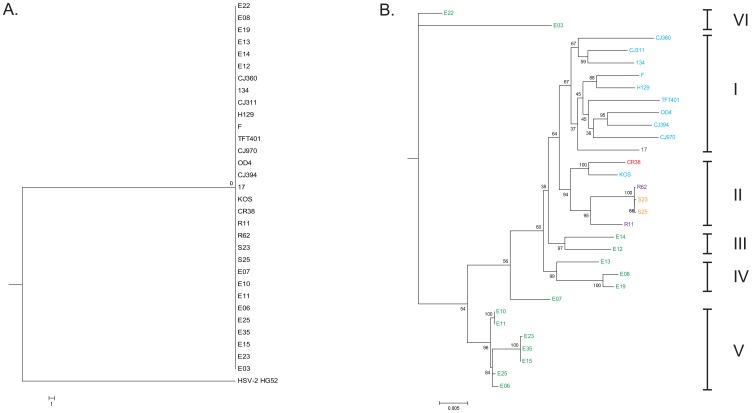
Phylogenetic trees featuring HSV-1 strains which depict the formation of six clades based on geographic origin. A maximum likelihood (ML) phylogenetic tree was constructed with 31 HSV-1 whole or partial genomic sequences, using HSV-2 as an outgroup. (B) An expansion of the HSV-1 specific node from the ML tree in (A). The ML tree was generated from aligned sequences using the Mega 5 package. Clade I includes European/North American strains, Clade II comprises East Asian strains and III, IV, V and VI are East African. HSV-2 was used as an outgroup. The viral isolates are colored according to country of origin and are as follows: U.S.A: light blue, U.K.: dark blue, China: red, South Korea: purple, Japan: orange, and Kenya: green.

The genomic sequences used for analysis were obtained from the NCBI Reference Database. The genomes of HSV-2 HG52 and 31 HSV-1 strains (Figure 1) were aligned with Clustal W [26] using Mega 5. [27] The mean genetic distances between HSV-1 and HSV-2, as well as between all HSV-1 strains were calculated using the maximum composite likelihood option with “complete deletion” of alignment gaps using Mega 5. Pairwise distances between all the HSV-1 and HSV-2 strains were calculated using the maximum composite likelihood option. Complete deletion of alignment gaps was performed when HSV-2 was compared to the HSV-1 strains as a group. Pairwise deletion was performed rather than complete deletion when comparing HSV-1 strains to each other in order to minimize overestimates of distance.

### Phylogenetic and Recombinational Analysis

Prior to phylogenetic analysis, gaps in the Clustal W genomic alignment were deleted, yielding 126,608 bp in the alignment. We performed maximum likelihood (ML) analysis on the genomic alignment using the RAxMLGUI package with the GTRCAT+I model and 500 replicates [28]. A phylogenetic network was then generated from the 500 bootstrap replicates using Splitstree 4 [29].

To address possible recombination between the viral strains, the alignments were broken up into twenty five, 5 kb partitions and one, 1.6 kb partition. The RAxMLGUI package was then used to analyze each of the twenty six partitions with the GTRCAT+I model and 500 bootstrap replicates. Utilizing Dendroscope 3 [30], a consensus tree was generated with a 70% confidence threshold from the 500 bootstrap replicates for each of the twenty six partitions. A consensus network was then assembled from the twenty six, 70% confidence threshold consensus trees with Splitstree 4.

### Estimated Divergence Times

The estimated divergence times of the HSV-1 and HSV-2 were calculated using the genomic alignment with the gaps deleted and the BEAST 1.7.4 software package [31]. First the substitution rates were allowed to vary along lineages using the uncorrelated lognormal relaxed clock model (UCLN) [32], using an exponential prior distribution with a mean of 5×10^−5^ substitutions/site/thousand years and an offset of 1×10^−7^, which yielded a 5% quantile value of 2.67×10^−6^, a median value of 3.5×10^−5^ and a 95% quantile value of 1.5×10^−4^. Additionally, the age of the Asian and European/North American population strain split was given a prior distribution with a mean of 34.0 thousand years and a standard deviation of 10.5 thousand years. Two BEAST runs were performed with 10 million generations. The resulting tree and log files were combined with LogCombiner v. 1.7.4 (http://beast.bio.ed.ac.uk/LogCombiner) with a burnin of 6 million for each run. The combined log and tree files were visualized by Tracer v. 1.5 (http://beast.bio.ed.ac.uk/Tracer). The resulting mean substitution rate was 1.34×10^−4^±6.4×10^−7^ substitutions/site/thousand years. BEAST analysis was run a second time with the UCLD model, an exponential prior distribution for substitution rates with prior mean 1E-4, and a prior age with a mean of 34.0 and a standard deviation of 5.5 to the Asian and European/North American population strain split. Two BEAST runs were performed with 10 million generations. The resulting tree and log files were combined with LogCombiner v. 1.7.4 with a burnin of 12 million. The combined log and tree files were visualized by Tracer v. 1.5 and Figtree v. 1.4 (http://beast.bio.ed.ac.uk/FigTree) respectively.

## Results

### Distance Analysis

The mean genetic distance between HSV-2 and HSV-1 was calculated at 23.16% using the maximum composite likelihood and complete deletion of gaps. The pairwise genetic distances between the HSV-1 strains ranged from 0% (E10 vs. E11) to 1.31% (CJ360 vs. E03), with a mean distance of 0.8%.

### Phylogenetic Analysis

To investigate the phylogenetic relationships of the available complete or nearly complete Herpes Simplex Type 1 genomic sequences, 1 HSV-2 and 31 HSV-1 sequences with origins from North America, Europe, East Africa and East Asia were obtained from NCBI (Table 1). The sequences were first aligned with Clustal W and a maximum likelihood (ML) tree (Figure 1) was generated. Figure 1A shows the initial ML tree, using HSV-2 as an outgroup and Figure 1B is an expansion of the HSV-1 specific node from the tree in Figure 1A. The resulting trees revealed a six clade pattern based on the geographic origin of the isolates. The European/North American viruses formed clade I, East Asian strains formed clade II and the East African viruses comprised clades III, IV, V and VI (Figure 1B). Only one virus did not sort according to geographic isolation and this was strain KOS, a North American derived strain which was placed in the East Asian clade II. While the East African strains E07 was placed into a node with clade IV viruses, it is genetically distant with low bootstrap values, thus we did not assign it to a clade.

**Table 1:** Genomes and accession numbers.

Species/Strain	Accession Numbers	City/State/Country of Origin	Source	Year ofIsolation	SequenceLength (b.p.)
*HSV-1*					
17	NC_001806	Glasgow, Scotland, UK	na	c. 1973	152,261
134	JN400093	Seattle, Washington, USA	Eye	a	149,697
CJ311	JN420338	Seattle, Washington, USA	Eye	a	150,153
CJ360	JN420339	Seattle, Washington, USA	Eye	a	147,074
CJ394	JN420340	Seattle, Washington, USA	Eye	a	148,466
CJ970	JN420341	Seattle, Washington, USA	Eye	a	149,127
CR38	HM585508	China	na	na	135,948
E03	HM585509	Kenya	na	na	135,658
E06	HM585496	Kenya	na	na	135,550
E07	HM585497	Kenya	na	na	135,520
E08	HM585498	Kenya	na	na	135,539
E10	HM585499	Kenya	na	na	135,510
E11	HM585500	Kenya	na	na	135,509
E12	HM585501	Kenya	na	na	135,577
E13	HM585502	Kenya	na	na	135,600
E14	HM585510	Kenya	na	na	135,588
E15	HM585503	Kenya	na	na	135,567
E19	HM585511	Kenya	na	na	135,775
E22	HM585504	Kenya	na	na	135,549
E23	HM585505	Kenya	na	na	135,558
E25	HM585506	Kenya	na	na	135,569
E35	HM585507	Kenya	na	na	134,296
F	GU734771	USA	na	na	152,151
H129	GU734772	San Francisco, California, USA	CNS	1977	152,066
KOS	JQ673480	Houston, Texas, USA	Lip	c. 1964	152,011
OD4	JN420342	Seattle, Washington, USA	Eye	a	150,381
R11	HM585514	South Korea	na	na	135,579
R62	HM585515	South Korea	na	na	135,544
S23	HM585512	Japan	na	na	135,003
S25	HM585513	Japan	na	na	135,676
TFT401	JN420337	Seattle, Washington, USA	Eye	a	151,912
*HSV-2*					
HG52	NC_001798	United Kingdom	Genital	1971	154,746

a Isolates were collected by Dr. John Chandler between 1975–1985.

**Figure 2:**
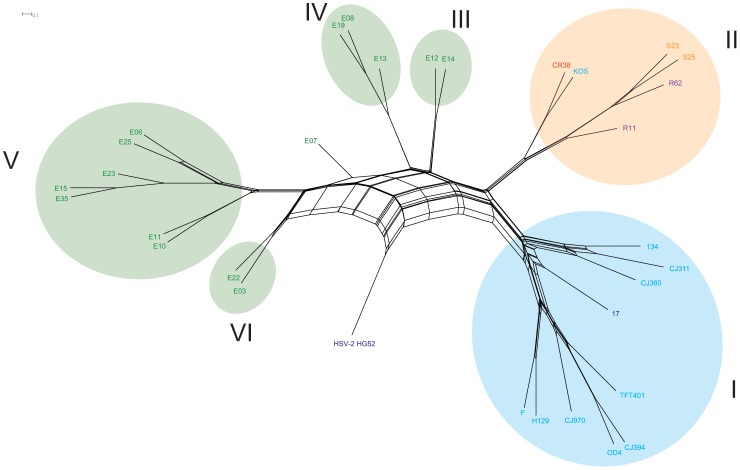
Phylogenetic network generated from 500, maximum likelihood bootstrap replicates. The HSV-1 strains in the network form the same six clades as in Figure 1. Clade I includes European/North American strains, Clade II comprises East Asian strains and III, IV, V and VI are East African. HSV-2 was used as an outgroup. Splitstree 4 was used to generate the network. The viral isolates are colored according to country of origin and are as follows: U.S.A: light blue, U.K.: dark blue, China: red, South Korea: purple, Japan: orange, and Kenya: green.

To examine the phylogenetic dissonance of the maximum likelihood analysis, a phylogenetic network was constructed using the 500 bootstrap replicate trees generated from the RAxML analysis (Figure 2). The six main phylogenetic clades were recovered and the isolated position of the African strain E07 was supported. The Eurasian clades I and II form one pole, while the African clades III, IV, V and VI form a continuum to the opposite pole.

### Recombinational Analysis

**Figure 3:**
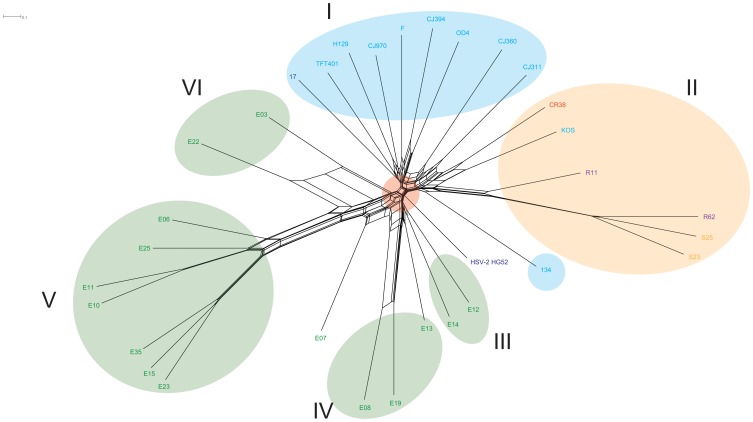
Consensus network constructed from 26 alignment partition consensus trees. The genome alignment was partitioned into 5(70% confidence threshold) was constructed for each partition, and a consensus network was generated from the combined results using Splitstree 4. Clade I includes European/North American strains, Clade II comprises North American/East Asian strains and III, IV, V and VI are East African HSV-2 was used as an outgroup. The viral isolates are colored according to country of origin and are as follows: U.S.A: light blue, U.K.: dark blue, China: red, South Korea: purple, Japan: orange, and Kenya: green.

To address possible recombination between the HSV-1 strains, the genomic alignment was broken up into 5 kb partitions. Each partition was then subjected to maximum likelihood analysis. A consensus tree with a 70% confidence threshold was generated from 500 bootstrap replicates for each partition. A consensus network was constructed by combining the consensus trees from each of the 26 partitions into a single file. The individual consensus trees are found in Figure S1. The resulting network is shown in Figure 3. This partition derived network closely resembles the unpartitioned network in Figure 2, however there are some key differences. The first difference is a recombination bottleneck between the Eurasian clades and the remaining African strains. The European/North American and Asian viruses form two distinct clades, however three North American strains (134, CJ311 and CJ360) were placed into the Asian clade II node. The connections to the clade II node are near to the node base, suggesting ancient recombination events. The network suggests recombination has occurred between; i) Asian clade II viruses KOS and CR38 ii) African clade VI strains E03 and E22 iii), African clade IV viruses E08 and E19.

### Molecular Clock

Following the initial phylogenetic tree analysis, we sought to estimate the relative divergence time for the strains in our analysis. Three preceding studies estimated the mutation rate of HSV-1 and herpes viruses in general to be 1.82×10^−8^ [22], 3×10^−8^ [33] and 3×10^−9^ [1] substitutions/site/year respectively. To determine what substitution rate fits best with the human population split data, BEAST analysis was performed using a wide substitution rate range, corresponding to 2.67×10^−9^ to 1.5×10^−7^ subs/site/year. A prior assumed that the HSV-1 European/North American and Asian clade split was 34,000±10,500 years BP so as to correspond with the human European and Asian population split 23–45 thousand years ago [34], [35], [36], [37]. The HSV-2 strain HG52 remained in the analysis as an outgroup. The BEAST analysis subsequently inferred an overall substitution rate of 1.34×10^−7^ (95% HPD upper: 2.14×10^−7^; 95% HPD lower: 7.48×10^−8^)subs/site/year. With the optimal substitution rate calculated, BEAST analysis was performed a second time with a prior mean substitution rate of 1×10^−7^ and an additional prior of 34,000±5,500 years BP. The BEAST analysis subsequently calculated an overall substitution rate of 1.38×10^−7^ (95% HPD upper: 1.89×10^−7^; 95% HPD lower: 9.5×10^−8^) subs/site/year. The resulting tree produced by BEAST is found in figure S2. The estimated divergence times are summarized in Table 2. Briefly, the estimated HSV-1 and HSV-2 divergence time was 2.18±0.753 million years BP, HSV-1 began to expand 50,300±16,700 years BP and the Eurasian strains diverged 32,800±10,900 years BP.

**Table 2:** Estimates of viral population divergence dates with respect to human populations splits.

Virus Strain Divergence	tMRCA	Human Population Split
HSV-1 and HSV-2	2.184±0.753 mya	Approx. advent of *Homo* [41]
HSV-1 strains	50.3±16.7 kya	Humans out of Africa ∼60 kya [39], [40]
Eurasian strains	32.8±10.9 kya	Asian-European: 20–40 kya [34], [35], [36], [37]
KOS and CR38	15.76±5.3 kya	Americas populated: 12–20 kya [64]

## Discussion

For the first time, a phylogenetic and recombinational analysis of all the available HSV-1 genomic sequences has been conducted. The results suggest that there are at least six clades of viruses with evidence for the possible existence of others. Our results also show that the clade structure is consistent with other data concerning human population structure and migration patterns and the results are also in agreement with the previous conclusion that HSV-1 has co-evolved with its host. [1].

### Genetic Distances

When we aligned the 31 HSV-1 sequences with Clustal W the mean genetic distance within the HSV-1 isolates was 0.8%. The mean genetic distance calculated here between HSV-1 and HSV-2 (23.16%) is lower than what has been reported previously. [38] This may be explained as an artifact due to the partial nature of several of the genomes in this study as well as the deletion of gaps within the aligned sequences. When the more fully sequenced HSV-1 strains from clade I were compared to HSV-2 using pairwise deletion, the distance increased to 27%. Therefore, the mean genetic distances between the HSV-1 strains reported here (0.8%) are likely an underestimate by approximately 15%, with the true number likely being about 0.92%.

### Phylogenetic Analysis

Maximum likelihood based phylogenetic analysis of the HSV-1 strains produced a six clade tree topology that correlated with the geographic origin of the isolate with one exception, HSV-1 KOS. Earlier phylogenetic work with single genes from the Unique Short region of the genome (US1, US4, US7 and US8) consistently produced a three clade pattern. [20], [21], [22] Recently, analysis with modest numbers of genomes from Europe and North Americans of European ancestry also yielded a three clade tree topology. [23] These results provide support for three sub-clades originating in Europe. Here, for the first time, a global sampling was used for a phylogenetic analysis. The topologic placement of the isolates in this study broke down along strict geographic lines (Figures 1 and 2); Europe/North America, East Asia, and Africa. This finding supports the hypothesis that Alphaherpesviruses co-evolved with their hosts [1] and the “out of Africa” theory of human evolution [39], [40]. The only strain that did not fit the geographic pattern was the North American derived strain KOS which broke the geographic topology pattern because it sorted into the East Asian clade II lineage. There are at least two potential explanations for the KOS lineage; it could represent recent global dissemination related to travel or, KOS may originally have been from the native Amerindian population. This is discussed more fully in the subsequent human migration section.

Our recovery of a six clade topology is not surprising and is likely temporary given the small number of sequences in the dataset. For example the East African strain E07 may represent a 7^th^ clade. The additional collection and sequencing of isolates other parts of the world, notably Western/Southern Africa, India, Melanesia, Central/South America, and Amerindian populations will probably yield new clades and may reveal firmer details of the history and migration patterns in these populations.

### Estimating Divergence Times

The observation that the HSV-1 viral strains sorted according to geographic origin and supported the “out of Africa” theory of human evolution suggested that a relaxed molecular clock could be applied to determine of date of divergence. Three previous estimates of either general herpesvirus or HSV-1 mutation rates have been reported as 1.82×10^−8^ [22], 3×10^−8^ [33] and 3×10^−9^ [1] substitutions/site/year. As such we sought to determine the substitution rate which best fit the human population divergence data. BEAST analysis was first performed with a wide substitution rate range, 2.67×10^−9^ to 1.5×10^−7^ subs/site/year and a prior (34,000±10,500 years BP) linking the viral European/North American and Asian strain split to that of the corresponding human strain split 23–45 thousand years BP [34], [35], [36], [37]. The resulting substitution rate was 1.34×10^−7^ subs/site/year, which is at least an order of magnitude greater than the previous estimates. It is unclear as to the discrepancy of substitution rates, however previous estimates examined only a small subset of genes as well as one or a small number of HSV-1 strains.

The BEAST analysis estimated an HSV-1 and HSV-2 divergence time of 2.184±0.753 million years BP. This time period corresponds roughly to the advent of the genus homo [41]. It unclear as to what would have precipitated the split between HSV-1 and 2, however a cognitive or behavioral change could be speculated as a cause.

### Recombination

Herpes simplex virus genomes are known to undergo high rates of recombination [42] and this can confound the phylogenetic analysis and the use of such data to calculate divergence times. However, most, if not all, of the data on HSV-1 recombination has been generated in laboratory settings where co-infection with large amounts of virus is used [43], [44], [45], [46], [47], [48], [49], [50], [51]. Such laboratory studies however, are highly artificial and it is not clear if these data can be extrapolated to natural infections.

There is little, if any, information available regarding how common recombination occurs in humans and there are several features of the natural history of HSV-1 that would act to reduce the chances of co-infection or superinfection with two different strains of virus. Transmission occurs by close contact, most commonly through infected saliva and is thus strongly interfamilial. Viral replication is restricted and localized to the site of infection and the innervating sensory nerve ganglia. The virus does not disseminate in its host. This reduces the number of infected cells available for recombination to occur. Viral replication subsides within a week or two and latent infection is established where a small percentage of neurons contain the virus and replication is suppressed. The low number of cells involved reduces the chances of co-infection of a single neuron with two strains of virus. Primary infection generates an adaptive immune response that suppresses replication and could reduce the probability of superinfection. Finally, expression of the viral glycoprotein D in cells renders them resistant to superinfection [52] and the latency-associated transcript (LAT), which is expressed in latently infected neurons interferes with superinfection [53]. When considered together, the probability of a circumstance that could lead to the generation of recombinant viruses could be quite low in natural infections.

To account for the potential effect of recombination one could identify regions of the viral genome that have not recombined but such regions are difficult to identify. In addition, as the number of strains available for analysis increases the probability of recombination free regions decreases. To date there do not appear to be significant hot spots for recombination and recombination appears to be random across the genome [43], [46], [54].

Bowden et.al. [55] sequenced 3 loci comprising 3% of the HSV-1 genome and reported high rates of recombination in a collection of strains from the United Kingdom or Korea. However, the use of single genes or small sets of genes can result in highly biased phylogenies that do not necessarily identify actual relationships. We took an alternative approach where we divided the genomes of the 31 isolates into twenty-five 5 KB segments and one 1.6 Kb segment. We then constructed 500 individual trees for each segment and then used these to generate a partition based network. The resulting network (Figure 3) suggests a recombination bottleneck, highlighted by a red circle, between the African and Eurasian strains. This finding supports an “out of Africa” model of human population spread, with limited back migration into Africa. The topology of the partition based network (Figure 3) closely resembles the ML bootstrap based network (Figure 2). The same six clades were recovered in the partition based network, however the topology between clades I and II was changed. The European/North American strains 134, CJ311 and CJ360 were clustered near the base of Asian clade II, which suggests ancient recombination events. Further analysis of the partition based network also indicated that the majority of recombination that was detected across the entirety of the tree occurred near the root nodes. Once the individual strains began diverging there was little evidence for recombination. The exceptions included; i) the African strains E03 and E22 ii)E08 and E19 and iii) KOS and CR38. Note that the recombination at the roots in the two European/North American groups occurred within the same cluster. This analysis suggests that recombination is not a confounding factor and can be accounted for in using HSV-1 genome sequences to study human populations.

Previous investigations examining recombination at the genomic level as well as with groups of single genes [22], [23], suggested that most if not all of the HSV-1 strains analyzed were recombinants and were genetic mosaics. The partitioned based network (Figures 3) presented here reinforces that conclusion. Recombination appeared to be random across the genome without obvious recombination hotspots or cold spots detected (data not shown).

### Relation to Human Migration

Other human pathogens such as JC virus [56], [57], [58] and *Helicobacter pylori* [59], [60], [61] have been shown to co-migrate and diversify with their human hosts. The phylogenetic tree data presented demonstrates that HSV-1 also does the same. HSV-1 establishes a latent, persistent infection and which enables it to easily travel with its host. While preliminary, our data raise the possibility that HSV-1 sequences could serve as a surrogate marker to analyze human migration and population structures. This would greatly facilitate such studies because viral isolates are easy to obtain and multiplex sequencing of viral genomes is much less costly than sequencing human genomes or SNP analysis. The HSV-1 genome is approximately 30 times larger than the JC virus genome, which may allow for finer genetic mapping due to a larger number of SNPs per genome.

The four clade structure recovered from the Kenyan samples shows the high level of diversity in HSV-1 sequence from this area and correlates with the genetic diversity of human populations in East Africa. No data was available from GenBank specifying the ethnic group from which the Kenyan samples were derived. It is tempting to speculate however that the four clades may be a result of the four major ethnic groups which have historically occupied this area of East Africa [62], [63]. Clade VI could be associated with hunter gatherer groups, which are thought to be the first to appear, clade V with Cushitic people, clade IV with Nilotic peoples and clade III with Bantu groups. The analysis of additional isolates could confirm these speculations and could further validate the use of HSV-1 in studying the history of human populations.

**Figure 4:**
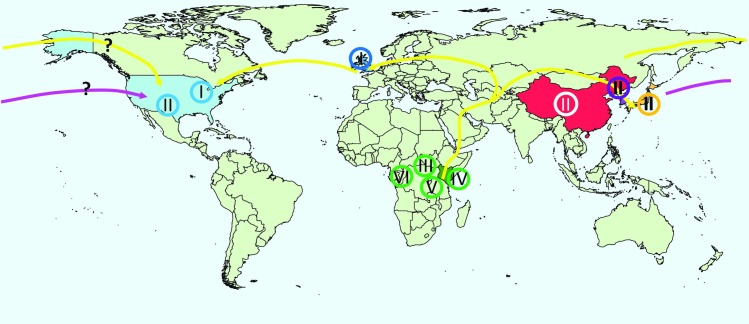
World map featuring the geographic location of the 6 HSV-1 clades with respect to human migration. The phylogenetic data supports the “out of Africa model” of human migration with HSV-1 traveling and diversifying with its human host. Each clade is depicted by a roman numeral inside a circle. Land migration is depicted by yellow lines and air/sea migration is shown by the pink line. The countries of origin for the strains in the current study are China (red), Japan (orange), Kenya (dark green), South Korea (purple), UK (dark blue), and USA (light blue). The map was generated using R (version 3.4.2, “maps” package).

The placement of North American derived strain KOS with the East Asian clade II was the only strain not to follow geographical lines. This could be due to access to modern travel or it could represent an indigenous Amerindian isolate. The BEAST analysis calculated an estimated divergence time of 15,760±5,300 (Table 2) between strain KOS and the Chinese virus CR38. This divergence time fits with the estimated time period in which the North American continent was populated from Asia, approximately 15,000 years BP [64]. As such we would propose that KOS is a representative of an Amerindian HSV-1 strain. A summary figure featuring the geographic location of the HSV-1 clades with respect to human migration is found in Figure 4.

In conclusion, for the first time global genome sequences from HSV-1 were subjected to phylogenetic and recombinational analysis. The results suggest the existence a minimum of six clades that sort according to the geographic origin of the strains. The recombinational analysis suggests that both intra- and inter-clade recombination have occurred. These results also suggest that sequencing and analysis of HSV-1 strains could serve as a surrogate marker to study human population structure and migration patterns.

## Supporting Information

Figure S1 Consensus trees (70% threshold value) for each of 5 kb partitioning of the genomic alignment.
(TIF)

Figure S2 Phylogenetic tree generated by BEAST. Height (95% HPD) bars are blue with a timescale at the bottom.
(TIF)
