# Correction: Using HSV-1 Genome Phylogenetics to Track Past Human Migrations

**DOI:** 10.1371/journal.pone.0217890

**Published:** 2019-05-30

**Authors:** Aaron W. Kolb, Cécile Ané, Curtis R. Brandt

## Notice of Republication

An incorrect version of Figure 4 was published in error. This article was republished on May 20, 2019 to correct for this error. Please download this article again to view the correct version.
