# Supplementary figures and images for: Using HSV-1 Genome Phylogenetics to Track Past Human Migrations
Source: PLoS One. 2013 Oct 16;8(10):e76267. doi: 10.1371/journal.pone.0076267 (PMC3797750; doi:10.1371/journal.pone.0076267)

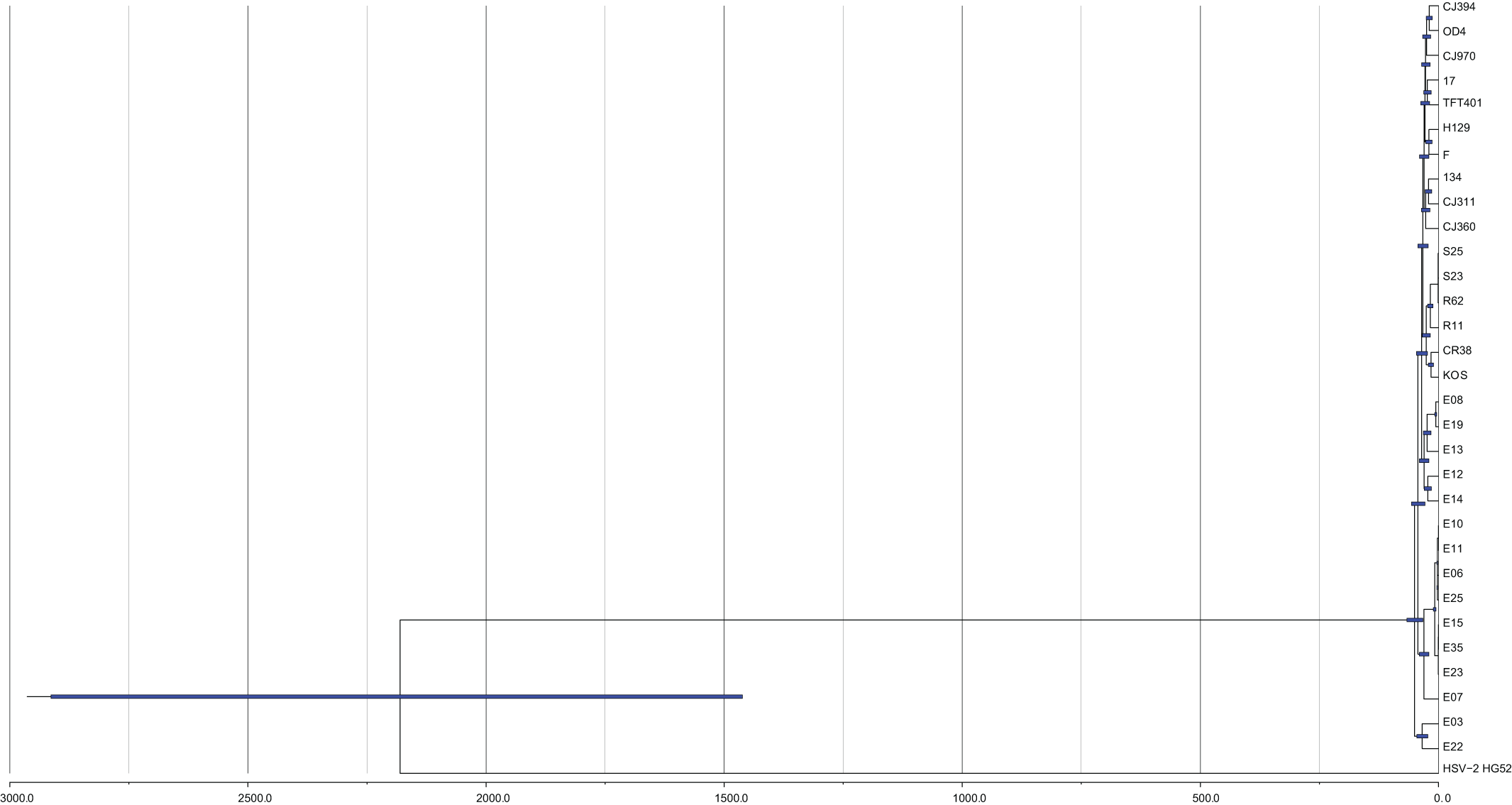

Supplement: Figure S2 — Phylogenetic tree generated by BEAST. Height (95% HPD) bars are blue with a timescale at the bottom. (TIF) [file pone.0076267.s002.tif]
